# Modified Anatomical Reconstruction of the Medial Collateral Ligament With Tibial Sling Bone Tunnels and Double-Bundle Semitendinosus Tendon

**DOI:** 10.1016/j.eats.2025.103625

**Published:** 2025-06-13

**Authors:** Sergio Marinho de Gusmão Canuto, José Leonardo Rocha de Faria, Igor Farias de Araújo, Pedro Enio Feitosa Bezerra, João Henrique da Silva Araújo, Diego Ariel de Lima, Diego Escudeiro de Oliveira, Bernardo Garcia Barroso, Arthur Macedo de Gusmão Canuto, Pedro Baches Jorge, Vitor Barion Castro de Pádua, Camilo Partezani Helito

**Affiliations:** a Ortoclínica, Orthopaedics Hospital, Maceió, AL – Brazil; b Knee Surgery Center, National Institute of Traumatology and Orthopedics (INTO), Rio de Janeiro, RJ – Brazil; c Research Division - National Institute of Traumatology and Orthopedics (INTO), Rio de Janeiro, RJ – Brazil; d National Institute of Traumatology and Orthopedics (INTO), Rio de Janeiro, RJ – Brazil; e Federal Rural University of Semiarid – Mossoró, RN – Brazil; f Sports Medicine department of Santa Casa of São Paulo – São Paulo, SP – Brazil; g Institute of Orthopaedic and Traumatology of Vitoria Apart Hospital, Serra, ES – Brazil; h Superior Study Center of Maceio (CESMAC) – Maceio, AL – Brazil; i Department of Orthopaedics and Traumatology Federal University of São Paulo, Paulist School of Medicine, São Paulo, SP – Brazil; j University of São Paulo – USP, São Paulo, SP – Brazil

## Abstract

This article describes a modification of an surgical technique for the anatomical reconstruction of the superficial medial collateral ligament (MCL) of the knee using a semitendinosus tendon graft in a minimally invasive approach. The MCL often is the site of severe injuries, which can result in long-term complications such as instability. The detailed surgical technique begins with an examination under anesthesia and diagnostic arthroscopy for accurate injury assessment. The semitendinosus tendon is used as a graft and fixed in the tibial communicating tunnels, with interference screw fixation only on the femur, restoring the integrity of the MCL. The postoperative protocol involves a careful rehabilitation process, including weight-bearing restriction and gradual return to sports activities. This technique offers advantages such as increased graft strength and lower surgical morbidity, making it a viable option for patients with anteromedial instabilities and grade III MCL injuries, especially when there are concomitant anterior cruciate ligament injuries. The anatomical reconstruction of the MCL with a double bundle and semitendinosus tendon graft is a promising technique that continues to be refined and studied in the quest for improved long-term outcomes in knee stability.

The medial collateral ligament of the knee (MCL) is the most commonly injured ligament.^1^^,^^2^ Its primary function is as the primary stabilizer against valgus stress of the knee, and it is also an important restrainer of external tibial rotation forces.^3^^,^^4^ The MCL usually heals in most isolated MCL injuries, with conservative treatment showing excellent results.^1^^,^^2^

In cases of complete isolated medial collateral ligament injury (grade III), failed conservative treatment, or associated ligament injuries, nonsurgical treatment of the MCL increases the risk of anterior cruciate ligament (ACL) rupture, posttraumatic osteoarthritis, and functional limitations.^5^ To avoid these outcomes, MCL reconstruction is chosen.^6^

Studies show that anatomical techniques have less biomechanical variation and result in better functional scores. Therefore, anatomical reconstruction is currently considered the most appropriate approach.^7^, ^8^, ^9^ In this Technical Note, we present a modification of a recently described an anatomical technique^10^ for the reconstruction of the superficial MCL that offers a valuable solution for patients facing these severe injuries. Our goal is to enhance this minimally invasive approach with the potential to reduce surgical morbidity and promote faster recovery.

## Surgical Technique

The patient is positioned in the supine position under spinal anesthesia. A cushion is placed under the hip joint of the limb that is undergoing operation. A pneumatic tourniquet is attached to the root of the thigh of the operated limb. A leg holder is used along with the pneumatic tourniquet, which allows for the application of valgus stress on the knee. Hair removal, asepsis, antisepsis, and draping are performed to begin the approach.

The procedure begins with the identification and resection of the ipsilateral semitendinosus tendon. Then, a 3- to 4-cm incision is made to remove the semitendinosus graft at the posterior one third of the anteromedial tibial face, 1 cm more posterior than usual because the same incision to remove the graft will be used to access the posteromedial crest of the tibia, about 6 to 5 cm below the joint line. The arthroscopic portals are created; thus, diagnostic investigation is carried out to identify possible associated intra-articular injuries. (Video 1).

**Fig 1:**
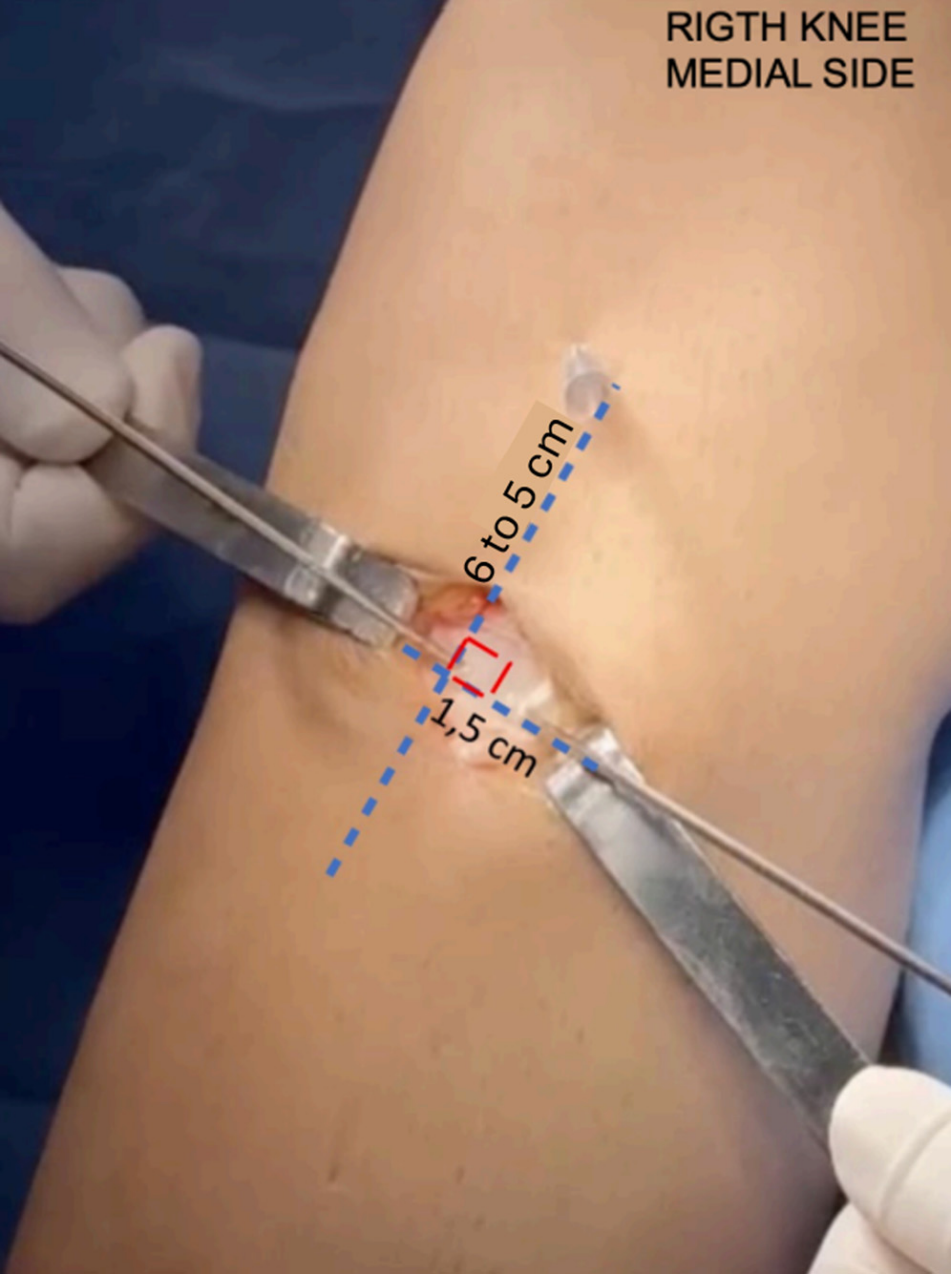
The patient is positioned in the supine position with the right knee hanging, showing a medial view of the knee, which displays positioning of the tibial tunnels at the level of the superficial medial collateral ligament insertion, approximately 6 to 5 cm form the joint line., with a 90° angulation and a distance of 1.5 to 1.8 cm between them.

A second 3-cm incision is made along the medial epicondyle of the femur. The structures are initially identified at their deep plane insertions. The MCL is identified at its femoral and tibial origins, marked with Kirschner wires. The femoral guide wire is positioned 3 mm posterior to the medial epicondyle of the femur. In the tibia, at the level of the superficial MCL insertion, about 6 cm for most people and 5 cm from the joint line in patients of shorter stature. Two Kirschner wires are passed at a 90° angle between them. The first is drilled from posteromedial to anterolateral, in the posteromedial crest of the tibia. The second wire is drilled from anteromedial to posterolateral on the medial face of the tibia at the same level. Then, with a 4.5- to 5-mm cannulated drill, depending on the thickness of the graft used, 2 converging tunnels are drilled in the tibia, maintaining a 90° angle, with a distance of approximately 1.8 cm to 1.5 cm between them (Fig 1, Video 1).

**Fig 2:**
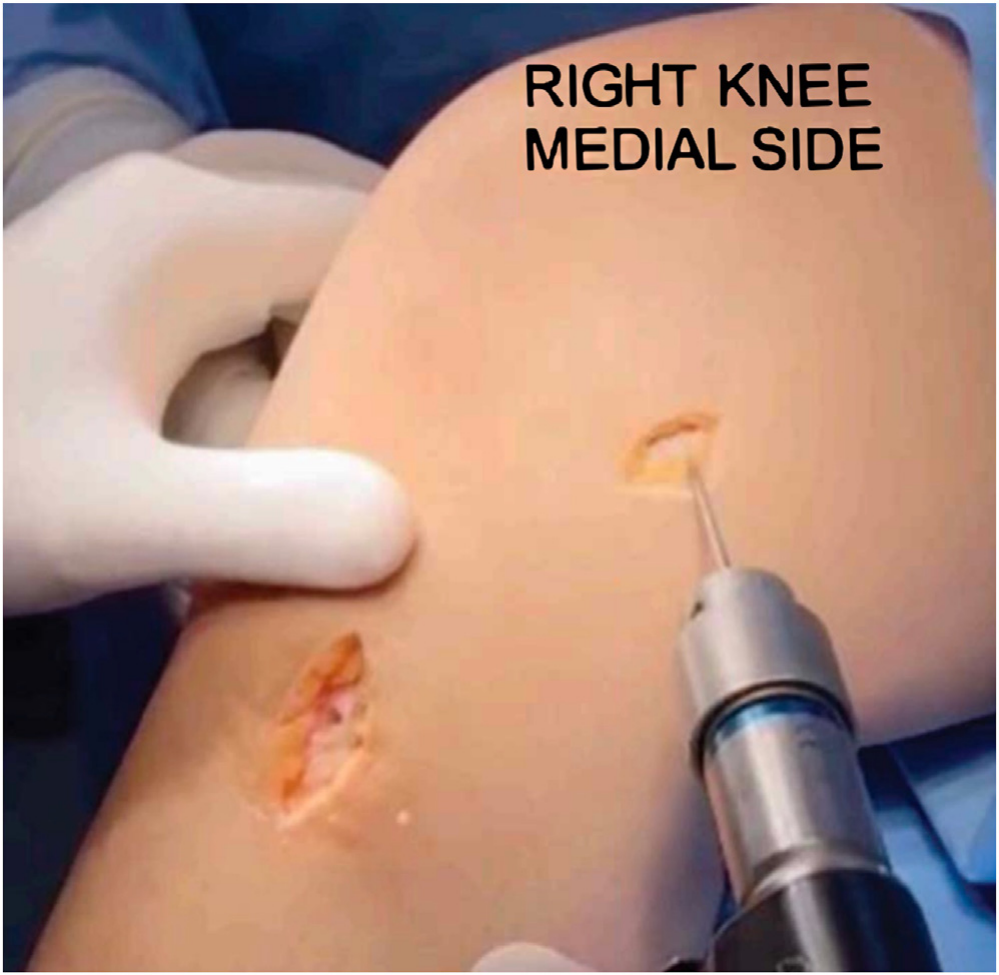
The patient is positioned in the supine position with the right knee hanging, showing a medial view of the knee, which displays positioning of the femoral tunnel 3 cm posterior to the medial epicondyle, from medial to lateral, with an angulation of 10 to 20° from distal to proximal.

**Fig 3:**
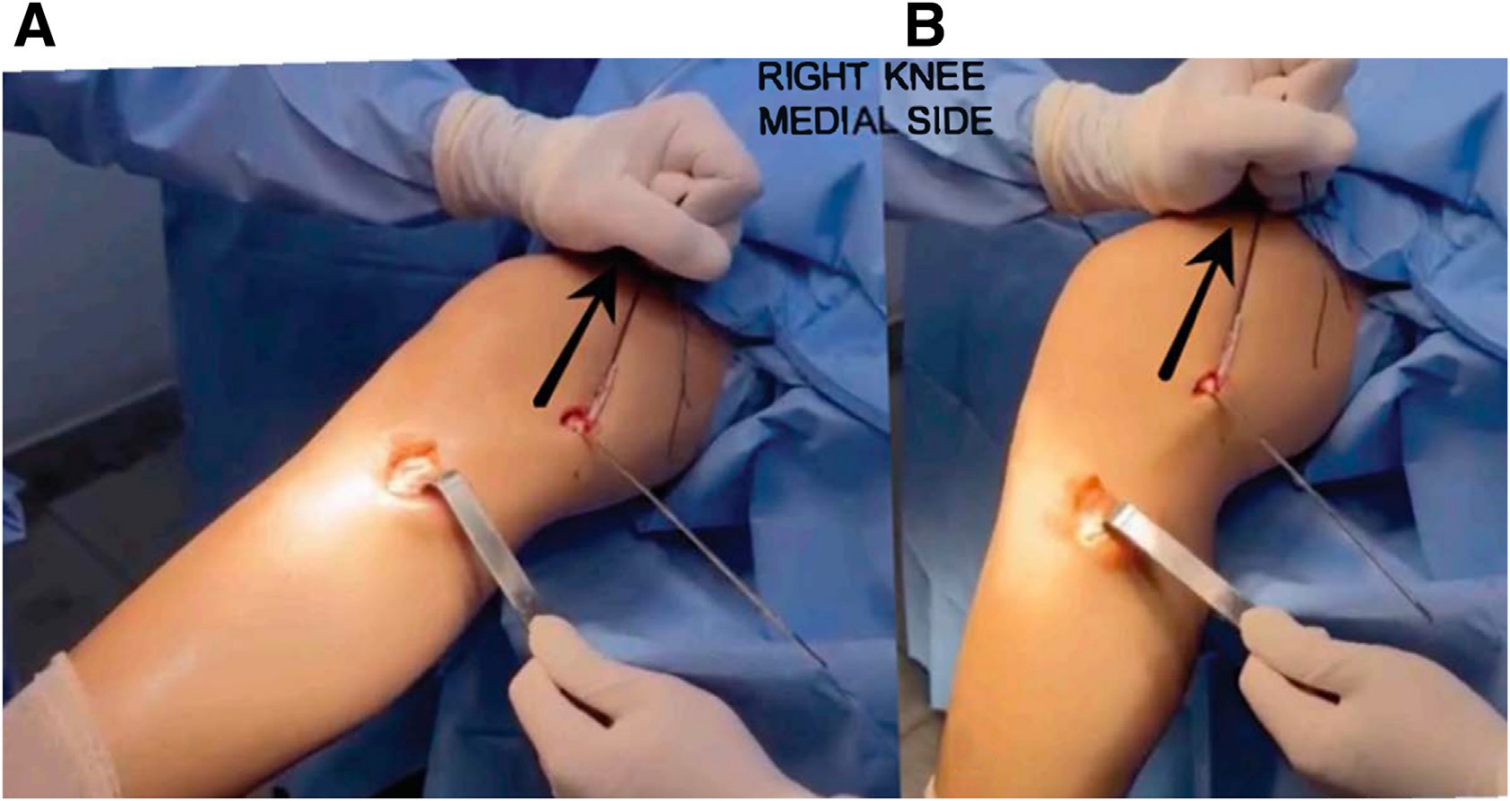
The patient is positioned in the supine position with the right knee hanging, showing a medial view of the knee, which displays graft-tying around the Kirschner wire to check isometry in the femur from 0 to 90° of movement associated with vertical traction.

We use the ETHIBOND thread (Ethicon) itself for the passage of the sutures through the tunnels, bending the needle into a U shape and using the noncutting side to pass through the tunnels, usually from posterior to anterior. Then, the semitendinosus graft is pulled through the tibial tunnels. Next, the femoral tunnel is drilled with a Kirschner wire 3 mm posterior to the medial epicondyle, from medial to lateral with an approximate 10 to 20° angle from distal to proximal (Fig 2). At this moment, we tie the graft around the Kirschner wire and check the isometry on the femur from 0 to 90° of movement (Fig 3). Confirming favorable isometry, we drill the femoral tunnel up to the lateral cortex with a 4.5-mm cannulated drill, passed through the previously positioned guidewire. Then, with a 6-mm drill, we drill to a depth of 50 mm (Video 1).

**Fig 4:**
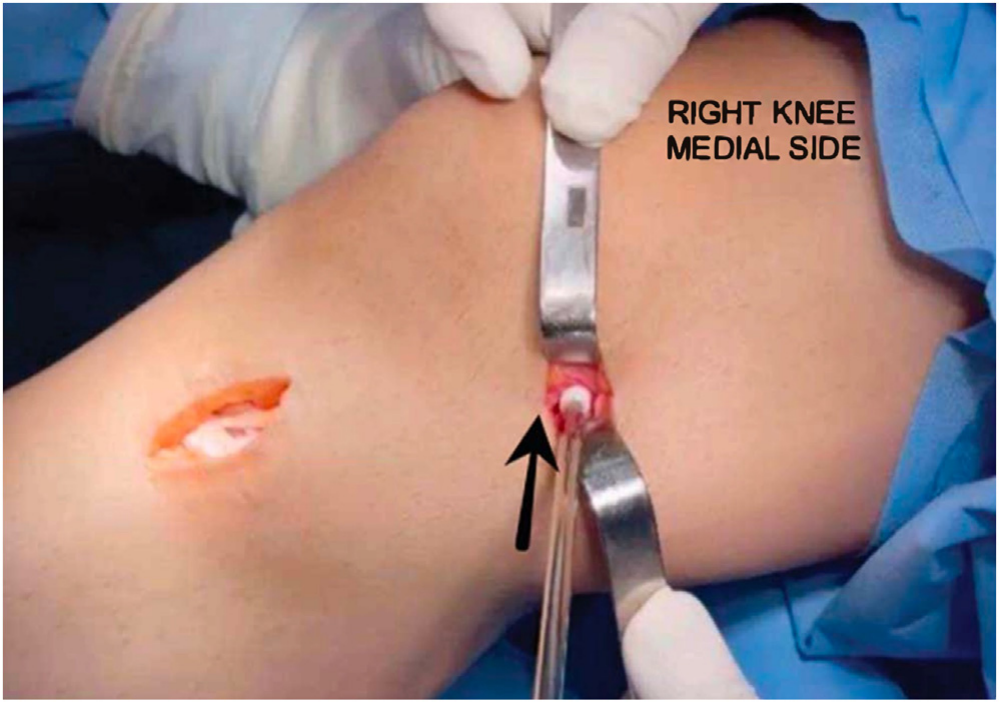
The patient is positioned in the supine position with the right knee hanging, showing a medial view of the knee, which displays fixation with axial traction at a 20° knee flexion angle in neutral rotation, applying varus force.

The ends of the graft are passed through the femoral tunnel and fixed at a 20° knee flexion angle in a neutral rotation, applying a varus force (Fig 4). The graft generally is fixed with a 7-mm bioabsorbable interference screw, which should be at least 1 mm larger than the femoral tunnel diameter (Video 1).

**Fig 5:**
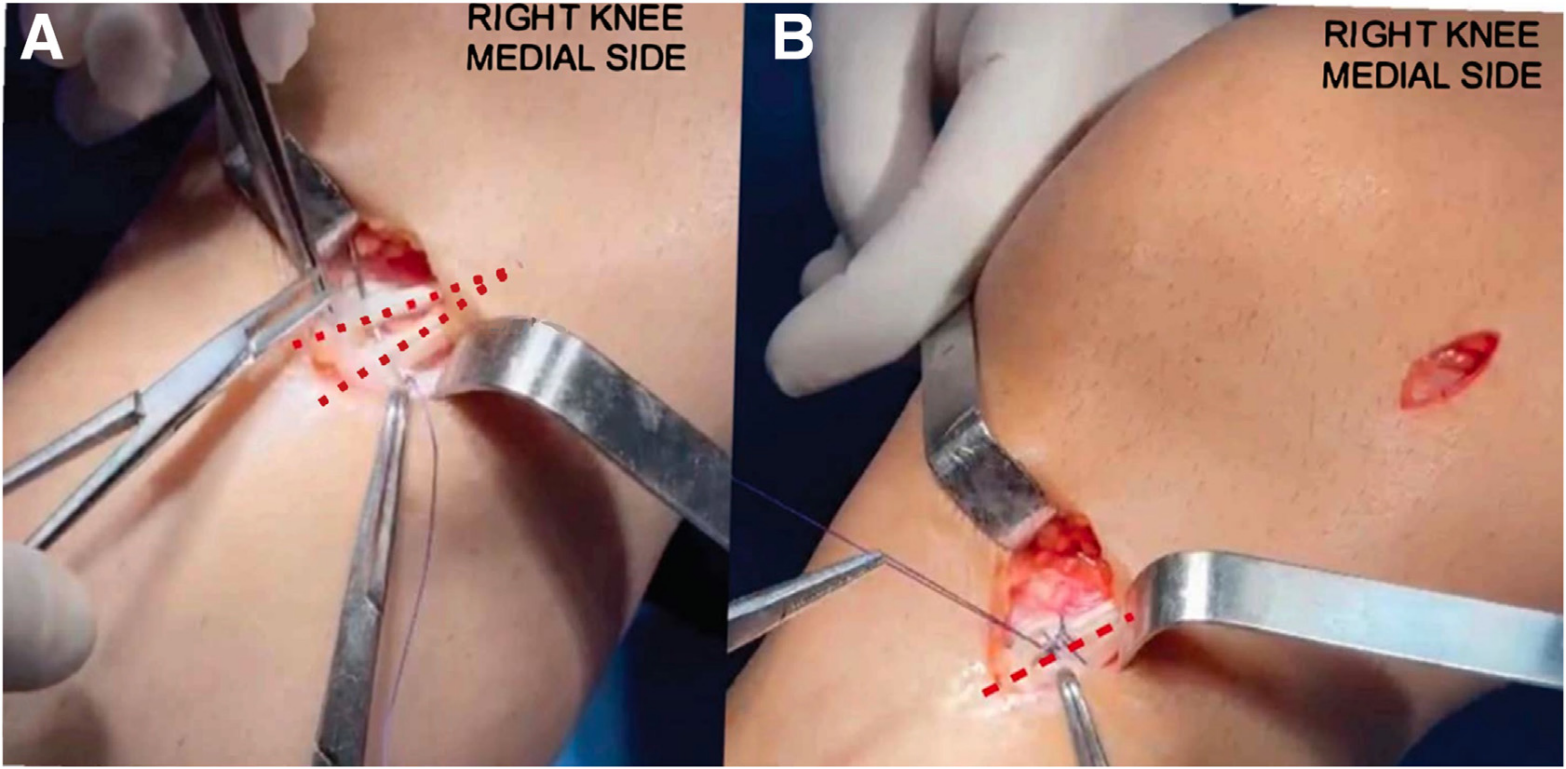
The patient is positioned in the supine position with the right knee hanging, showing a medial view of the knee, which displays suturing of the 2 distal ends of the semitendinosus along with the remnant of the superficial medial collateral ligament.

After the complete fixation of the MCL, we suture and approximate the 2 distal ends of the semitendinosus along with the remaining superficial MCL for about 2 to 3 cm proximally, ensuring greater graft tension and a tenodesis in the superficial MCL, improving rotational and valgus stress control (Fig 5). Finally, the tissue closure is performed in layers, preserving the appropriate layer in which the MCL should be positioned (Video 1).

### Postoperative Protocol

In the postoperative period, we do not use braces. Patients are restricted to partial weight-bearing. We keep them non−weight-bearing for 4 weeks and partial weight-bearing for another 2 weeks. The main goal until the sixth week is to achieve passive range of motion from 0 to 90° and complete knee extension with good quadriceps activation. Between 6 and 12 weeks, full weight-bearing and range of motion without a knee immobilizer are allowed. At 3 months, patients can run in a straight line. A gradual return to sports activities is allowed at 5 months for nonpivoting sports, at 6 months for noncontact pivoting sports, and at 8 months for contact pivoting sports.

## Discussion

The surgical technique described offers several significant benefits for patients with severe MCL injuries of the knee. First, the minimally invasive approach employed reduces surgical morbidity, allowing for quicker recovery and potentially resulting in fewer postoperative complications, such as joint stiffness. The technique aims to restore knee stability more isometrically, which can improve the long-term efficacy of the reconstruction, helping prevent complications such as osteoarthritis and functional limitations.^9^, ^11^, ^12^ The choice to use a double bundle of the superficial MCL increases the graft's strength and, therefore, the robustness of the reconstruction.^13^^,^^14^ In addition, the absence of implants for tibial side graft fixation potentially reduces surgical time and costs, making this technique a viable option for patients with multiligament injuries.^15^, ^16^, ^17^

A prospective study conducted by LaPrade and Wijdicks^18^ presents an approach to anatomical medial knee reconstruction, focusing on the MCL and posterior oblique ligaments. This research followed 28 patients undergoing this surgical technique, with the authors observing significant improvement in overall patient function compared with pre- and postoperative scores. The authors emphasize the importance of anatomical MCL reconstruction for more isometric restoration and better long-term efficacy.^19^ Our article describes a technique using a semitendinosus tendon graft, emphasizing minimizing surgical morbidity and potentially faster recovery.

The study by Mowers et al.^20^ highlights the importance of the choice between reconstruction and repair of the MCL in patients with medial injuries. Although both procedures have shown improvements in knee evaluation scores, such as the Lysholm and the Tegner score, MCL repair exhibited greater rates of postoperative knee stiffness and failures compared with MCL reconstruction during a minimum follow-up period of 2 years.^21^ These findings emphasize the importance of our technique, which has the potential for quicker recovery and less surgical trauma, potentially reducing postoperative complications such as joint stiffness.

Our study uses a double-bundle technique for superficial MCL reconstruction, promoting a minimally invasive approach with the potential for less surgical morbidity and faster recovery. The study by Lind et al.^7^ investigated the clinical outcomes of an MCL reconstruction technique in patients with chronic instability. The results also demonstrated significant improvements in medial knee stability, patient satisfaction, and quality of life after surgery.

Because of the high healing potential of the MCL, the need for surgical intervention is rare.^22^ Surgical treatment should be considered in chronic injuries presenting symptomatic residual instability.^7^^,^^8^ Clinically, an isolated grade III MCL injury is rare, and the risk of concomitant ligament injury is 78%.^20^^,^^22^ In cases of concomitant ligament injuries, 95% involve the ACL.^16^ For patients with concomitant ACL injuries and grade III MCL injury, surgical treatment should be performed.^23^^,^^24^ Our technique was also developed to be used in these cases.

Despite advances in understanding and improving MCL reconstruction techniques, there is still no consensus on the standard procedure, mainly because of the lack of clinical evidence regarding the outcomes of these procedures.^25^ Because the superficial MCL is the primary medial static stabilizer of the knee, the treatment should aim to correctly place the ligament in its anatomical insertion for a more isometric reconstruction. Therefore, changes in graft length should be avoided because this may limit the range of motion and increase graft tension, leading to long-term reconstruction failure and increased compression load in the medial compartment.^9^^,^^11^^,^^15^

As a limitation of the technique, patients of smaller stature, grafts may be shorter in length, requiring tibial tunnels to be placed more proximally generally around 5 cm or less distal to the joint line. In Table 1, the advantages and disadvantages of this technique are summarized. We believe that the technique described in this article for MCL reconstruction is capable of restoring medial knee stability in most patients through a less-invasive, easily reproducible, and technically less challenging approach, presenting a faster learning curve than currently available techniques.

**Table 1:** Advantages and Disadvantages of MCL Reconstruction With Tibial Sling Tunnels

Advantages of MCL Reconstruction With Tibial Sling Tunnels	Disadvantages of MCL Reconstruction With Tibial Sling Tunnels
Single-screw fixation: Uses only one screw to secure the entire system, simplifying the procedure and reducing the need for additional hardware.	Risk of graft damage: If the graft is introduced at an incorrect angle, the screw can cut into the graft, potentially compromising the reconstruction
Enhanced healing potential: The use of double tibial tunnels increases the surface area for graft integration, potentially improving the healing process.	Risk of tunnel wall fracture: If a fracture occurs in the wall between the double tibial tunnels, it can lead to the failure of the entire fixation, necessitating revision surgery.
Single graft requirement: The procedure requires only one graft, reducing the complexity and potential morbidity associated with harvesting multiple grafts.	Graft length limitation: If the graft is shorter than the optimal length (typically less than 23 cm), it can complicate femoral fixation, making the procedure more challenging.
Double-bundle graft: The double-bundle technique provides greater rotational stability and strength, improving overall knee stability.	Increased technical demands: The use of double-bundle grafts may require more precise surgical technique, increasing the complexity of the procedure

MCL, medial collateral ligament.

## Disclosures

All authors (S.M.d.G.C., J.L.R.d.F., I.F.d.A., P.E.F.B., J.H.d.S.A., D.A.d.L., D.E.d.O., B.G.B., A.M.d.G., P.B.J., V.B.C.d.P., C.P.H.) declare that they have no known competing financial interests or personal relationships that could have appeared to influence the work reported in this paper.

## Supplementary Data

Video 1 Approach: With the patient already positioned and having performed the valgus stress maneuver to confirm the injury to the medial collateral ligament (MCL), in addition to the basic arthroscopic evaluation, a 3- to 4-cm incision is made to remove the semitendinosus graft from the posterior third of the anteromedial aspect of the tibia, approximately 5 cm below the joint line, allowing for visualization of the tendon and the posteromedial ridge of the tibia. After identifying and isolating the tendon, the procedure continues with the removal and preparation of the tendon. Tibial tunnels: At the tibia at the level of the superficial MCL insertion, approximately 5 cm from the joint line, 2 Kirschner wires are inserted. The first is drilled from posteromedial to anterolateral, on the posteromedial ridge of the tibia. An angle of 90° should be maintained with a distance of approximately 1.8 to 1.5 cm between them. The second wire is drilled from anteromedial to posterolateral on the medial face of the tibia at the same level. Subsequently, using a 4.5- to 5-mm cannulated drill, depending on the thickness of the graft used, the 2 converging tunnels are drilled, guided by the Kirschner wires. Femoral tunnel and graft passage: Subsequently, a second 3-cm incision is made along the medial epicondyle of the femur. The femoral tunnel is drilled with a Kirschner wire positioned 3 mm posterior to the medial epicondyle, from medial to lateral with an approximate angulation of 10 to 20° from distal to proximal. Subsequently, the femoral tunnel is drilled to the lateral cortex with a 4.5-mm cannulated drill passed over the previously positioned guide wire, followed by a 6-mm drill to a depth of 50 mm. Afterwards, a needle loaded with thread is prepared for passage through the tibial tunnels, serving as a transport thread for the graft. The graft is then passed and must be tied around the Kirschner wire to check isometry in the femur from 0 to 90° of movement, confirming favorable isometry. Graft fixation: With the graft extremities already passed through the femoral tunnel, fixation is executed at a knee flexion angle of 20° in neutral rotation, applying a varus force. The graft is then secured with an interference screw that should be at least 1 mm larger than the diameter of the femoral tunnel. The final surgical step is the suturing to approximate the 2 distal strands of the semitendinosus along with the remnant of the superficial medial collateral ligament, in order to increase the tension in the graft and achieve a "tenodesis" with the superficial MCL, enhancing rotational control and valgus stress resistance. Checking: After the reconstruction, pretensioning of the graft should be performed intraoperatively with cycling associated with valgus stress.
